# Induction of epithelial to mesenchymal transition in PMC42-LA human breast carcinoma cells by carcinoma-associated fibroblast secreted factors

**DOI:** 10.1186/bcr1656

**Published:** 2007-02-20

**Authors:** Stephanie C Lebret, Donald F Newgreen, Erik W Thompson, M Leigh Ackland

**Affiliations:** 1 Deakin University, Burwood Highway, Burwood, Melbourne, 3125, Australia; 2 The Murdoch Children's Research Institute, Flemington Road, Parkville, Melbourne, 3050, Australia; 3 Department of Surgery, University of Melbourne, Grattan Street, Parkville Melbourne, 3050, Australia; 4 St. Vincent's Institute of Medical Research, Victoria Parade, Fitzroy, Melbourne, 3065, Australia; 5 Bernard O'Brien Institute for Microsurgery, Fitzroy Street, Fitzroy, Melborune, 3065, Australia

## Abstract

**Background:**

Breast carcinoma is accompanied by changes in the acellular and cellular components of the microenvironment, the latter typified by a switch from fibroblasts to myofibroblasts.

**Methods:**

We utilised conditioned media cultures, Western blot analysis and immunocytochemistry to investigate the differential effects of normal mammary fibroblasts (NMFs) and mammary cancer-associated fibroblasts (CAFs) on the phenotype and behaviour of PMC42-LA breast cancer cells. NMFs were obtained from a mammary gland at reduction mammoplasty, and CAFs from a mammary carcinoma after resection.

**Results:**

We found greater expression of myofibroblastic markers in CAFs than in NMFs. Medium from both CAFs and NMFs induced novel expression of α-smooth muscle actin and cytokeratin-14 in PMC42-LA organoids. However, although conditioned media from NMFs resulted in distribution of vimentin-positive cells to the periphery of PMC42-LA organoids, this was not seen with CAF-conditioned medium. Upregulation of vimentin was accompanied by a mis-localization of E-cadherin, suggesting a loss of adhesive function. This was confirmed by visualizing the change in active β-catenin, localized to the cell junctions in control cells/cells in NMF-conditioned medium, to inactive β-catenin, localized to nuclei and cytoplasm in cells in CAF-conditioned medium.

**Conclusion:**

We found no significant difference between the influences of NMFs and CAFs on PMC42-LA cell proliferation, viability, or apoptosis; significantly, we demonstrated a role for CAFs, but not for NMFs, in increasing the migratory ability of PMC42-LA cells. By concentrating NMF-conditioned media, we demonstrated the presence of factor(s) that induce epithelial-mesenchymal transition in NMF-conditioned media that are present at higher levels in CAF-conditioned media. Our *in vitro *results are consistent with observations *in vivo *showing that alterations in stroma influence the phenotype and behaviour of surrounding cells and provide evidence for a role for CAFs in stimulating cancer progression via an epithelial-mesenchymal transition. These findings have implications for our understanding of the roles of signalling between epithelial and stromal cells in the development and progression of mammary carcinoma.

## Introduction

In the mammary gland, the extracellular matrix (ECM) influences cell growth, migration, morphology, proliferation, differentiation and biosynthetic activities [1]. Stromal-epithelial interactions in the mammary gland also play an important role in cancer development. This so-called reactive stroma differs from the stroma of the normal mammary gland, exhibiting changes similar to those seen with wound healing, such as fibroblast proliferation and extracellular matrix remodelling, although in an uncontrolled manner [2]. The stromal-epithelial interactions between fibroblasts and the luminal and myoepithelial cells of the mammary gland are important for normal development [3], and it has been postulated that cancer may be a physiological response to abnormal extracellular environment, and that disruptions in stroma-epithelium reactions may accelerate carcinogenesis [4-8]. Conversely, normal stroma can efficiently inhibit the expression of characteristics of neoplasm [4]. Most notably, it has previously been demonstrated that irradiation of the cleared mammary fat pad of mice causes malignant progression of transplanted normal mammary cells [9].

Fibroblasts produce stromal ECM proteins [10] and secrete many growth factors and hormones, including hepatocyte growth factor, insulin-like growth factor (IGF)-I, IGF-II, epidermal growth factor (EGF), transforming growth factor (TGF)-α, TGF-β_1_, TGF-β_2_, TGF-β_3_, fibroblast gowth factor (FGF)-7, FGF-2, FGF-10 and interleukin-6 [10]. Fibroblasts are also involved tumourigenesis, through the synthesis, deposition and remodelling of the ECM that occurs in mammary carcinoma, and are also the main source of paracrine factors that influence the growth of carcinoma cells [10]. *In vitro *culture and *in vivo *tissue recombination systems demonstrate that factors derived from tumour fibroblasts stimulate tumour progression of nontumourigenic epithelial cells [11,12].

Breast carcinoma is accompanied by changes in both the acellular and cellular components of the microenvironment, the latter typified by a switch from normal mammary fibroblasts (NMFs) to myofibroblasts during cancer progression. Myofibroblasts, also known as cancer-associated fibroblasts (CAFs), have smooth muscle phenotypic properties [13], typified by the expression of α-smooth muscle actin (SMA) *in vivo *and *in vitro*. [1] In primary cultures from normal human breast tissue, there are few or no α-SMA-positive stromal cells, although high frequencies have been observed in primary cultures from breast carcinomas. CAFs also express fibroblast activation protein (FAP), a 93 kDa cell surface protein of reactive-tumour stromal cells that is not present in most normal human adult tissue.

CAFs were first identified over 30 years ago in healing rat wounds [14], and may influence the propensity of luminal epithelial cells to undergo an epithelial-mesenchymal transition (EMT) and hence become malignant [15]. CAFs differ from NMFs also in their increased expression of growth factors [16,17], and different profiles of ECM molecule synthesis [18-20] and ECM-altering proteases and protease inhibitors [21,22]. The role played by fibroblasts in the development or progression of cancer is not yet fully understood, and the use of physiologically relevant co-culture models using fibroblasts from normal and cancer stroma may provide a tool for the analysis of the effect of fibroblasts.

PMC42-LA is a heterogeneous human breast cancer cell line with stem cell-like properties [23,24]. When cultured within a reconstituted extracellular matrix (Engelbreth-Holm-Swarm sarcoma [EHS] matrix) PMC42-LA cells form hollow, alveolar-like structures, or organoids, that express β-casein in the presence of lactogenic hormones [25]. In the presence of EGF, PMC42-LA cells in two-dimensional culture undergo an EMT [25,26]. Depending on the tissue culture environment, PMC42-LA can exhibit luminal epithelial or myoepithelial markers [27]. These inducible features of PMC42-LA, in addition to the many breast-like characteristics originally described [23,24,26,28], make PMC42-LA an attractive cell culture model for investigation of the differential effects NMFs and CAFs.

We previously showed that fibroblasts induce a myoepithelial switch in PMC42-LA cells, with primary mammary fibroblasts being more effective than immortalized skin fibroblasts [27]. In the present study we investigated the differential effects of CAFs compared with NMFs on the phenotype and behaviour of PMC42-LA cells and organoids. Our results provide direct evidence for a role for CAFs in contributing to cellular disorganization and loss of cell-cell adhesion, and demonstrate that CAFs, as compared with NMFs, significantly increase the migratory/invasive ability of PMC42-LA cells.

## Materials and methods

### Cell culture

PMC42-LA cells were grown at 37°C in RPMI-1640 medium with 10% (vol/vol) foetal bovine serum (FBS; Thermo Trace, Melbourne, Australia). Primary human NMFs were isolated from human breast tissue with appropriate consent from women undergoing reduction mammoplasty, in accordance with the standards of St. Vincent's Hospital Human Ethics Committee. Primary mammary CAFs were obtained from minced tumour tissue of a mammary tumour biopsy. Tissue was minced and digested with 200 ml collagenase (Sigma-Aldrich, St. Louis, MO, USA) and 100 ml hyaluronidase (Sigma-Aldrich) in Dulbecco's modified Eagle medium (DMEM)/Ham's F-12 (1:1) with 10% (vol/vol) FBS containing penicillin, streptomycin and amphoporicin B (Thermo Electron Corporation, Melbourne, Australia) overnight at 37°C and 5% carbon dioxide with gentle rocking. Digested tissue was centrifuged at 600 *g *for 10 min, and the cell pellet was resuspended in medium and filtered through a 40 μm cell strainer. The cells were allowed to attach overnight, cultured in growth medium as above, and used within 10 passages. SV40 immortalized human skin fibroblasts (GM847), obtained for diagnostic purposes, were grown and maintained in basal medium (Eagle's) with 10% (vol/vol) FBS (Sigma-Aldrich) for use as a control. Cells were passaged in 0.05% trypsin/EDTA (Sigma-Aldrich) when confluent; primary cells were passaged a maximum of 11 passages.

To induce organoid formation of PMC42-LA cells, 150 μl undiluted EHS (Sigma-Aldrich) was spread with a cell scraper on chilled PET track-etched/porous membrane cell culture inserts (0.4 μm pore size; Becton Dickinson Labware, Franklin Lakes, NJ, USA). The filters were incubated at 37°C for 30–45 min to allow the EHS to set. Once set, 150 μl diluted (4% vol/vol in chilled distilled water) EHS was placed on the surface of the set EHS, followed by 2 ml of RPMI 10% FBS (ThermoTrace) containing 10^6 ^PMC42-LA cells. Then, 3 ml RPMI 10% FBS was placed beneath the filter using a pipette. Cells were incubated for 5 days at 37°C with 5% carbon dioxide before processing. For EHS matrix with fibroblasts beneath the filter, the same procedure was followed except that 0.5 × 10^5 ^previously trypsinised fibroblasts (NMFs or CAFs) were placed in the well beneath the filter insert with 1 ml DMEM/Ham's F-12 (10% FBS; Thermo Electron Corporation) and 2 ml RPMI (Thermo Trace).

### Conditioned medium

For conditioned medium experiments, attached confluent fibroblasts were rinsed in phosphate-buffered saline (PBS), and fresh DMEM:Ham's F-12 (10% FBS) media added to flasks. Flasks were returned to 37°C with 5% carbon dioxide, and after 48 hours the conditioned medium was collected, double filter sterilized using an eccentric tip syringe (Terumo Corporation, Tokyo, Japan) and sterile nonpyrogenic 0.2 μm filter (Schleicher & Schuell BioScience, Keene, NH, USA), and used at a 1:1 ratio in addition to fresh RPMI on top or beneath the filter.

### Media concentration

Either 10 ml (1×), 5 ml (2×), 2.5 ml (4×), or 1 ml (10×) fresh DMEM:Ham's F-12 (10% FBS) was added to confluent NMFs. After 24 hours, medium was collected and used to culture PMC42-LA cells on glass cover slips before scratch wound assays.

### Cell counts

Cell counting was conducted using a standard haemocytometer, and cell viability tests were conducted using dye exclusion with trypan blue solution (0.4%; Sigma-Aldrich).

### Migration assay

Nitric acid-treated glass cover slips were used to culture PMC42-LA cells in six-well plates (Nunc, Roskilde, Denmark) for scratch tests. Using a sterilized plastic cafeteria fork, a series of scratches were made on confluent cover slips and these were processed for indirect immunocytochemistry as described below. For each cover slip, four scratches were made, averaging between 367 μm (minimum) and 392 μm (maximum) width, at time 0. Each scratch was measured at several time points and averaged, and the extent of wound closure was measured microscopically, compared with the original width of scratches and averaged. Data were processed and standard deviation calculated using Excel (Microsoft Corp., Redmond, WA, USA).

### Antibodies

Mouse anti-human β-actin, anti-human catenin-β and goat anti-human vimentin antibodies were purchased from Sigma-Aldrich. Mouse anti-human vimentin and mouse anti-human E-cadherin antibodies were purchased from Zymed laboratories, Inc. (San Francisco, CA, USA) and mouse anti-human α-SMA antibodies were purchased from Dako (Denmark, Europe). Goat anti-human E-cadherin and rabbit anti-active caspase-3 antibodies were purchased from Chemicon International Inc. (Temecula, CA, USA) and rabbit anti-human α-SMA antibodies from Abcam plc. (Cambridge, UK); anti-human FAP antibodies (muF19) from the biological production facility at Austin Hospital were kindly donated by the Ludwig Institute of Cancer Research (Heidelberg, Australia). The Alexa Fluor secondary antibodies used for immunocytochemistry were purchased from Molecular Probes Inc. (Eurgene, OR, USA) and included goat anti-mouse IgG labelled with Alexa 488, donkey anti-sheep IgG labeled with Alexa 594 and donkey anti-rabbit IgG labelled with Alexa 594. Anti-mouse horse-radish peroxidase conjugated antibody from Sigma-Aldrich was used as a secondary antibody in Western blot analysis.

### Protein extraction and quantification

Confluent PMC42-LA cells cultured in flasks were treated with 0.05% (vol/vol) trypsin/EDTA solution (Sigma-Aldrich), cell suspensions were centrifuged and the pellet re-suspended in 5 ml PBS. Following two washes in PBS and re-centrifugation, the pellets were stored at -80°C until needed, or prepared for quantification as described.

PMC42-LA cells cultured in EHS to form organoids were washed twice in PBS, following which 2 ml of 5 mg/ml dispase (Sigma-Aldrich; 20 mg/ml stock concentration) in RPMI/10% FBS was added to the filters. The filters were incubated for 30 to 90 min, or until cells were detached. Cells were centrifuged at 3,000 rpm for 5 min, washed twice with PBS, and the pellet was dried and then stored at -80°C.

Cell pellets were re-suspended in 500 μl of 1% (weight/vol) SDS in 10 mmol/l Tris-HCl (pH 7.5) on ice and homogenized by passaging through a 21-guage needle 15 times on ice, after which cells were sonicated (40% power output, 30% duty cycle) twice using a Microson Ultrasonic cell disrupter (Misonix Incorporated, Farmingdale, NY, USA). Samples were centrifuged and the supernatant quantified using the Pierce BCA Protein Assay Reagent Kit (Perbio, Rockford, IL, USA), in accordance with the manufacturer's instructions. The protein concentrations were determined by measuring the absorbance at 595 nm.

### SDS-PAGE and Western blot analysis

Proteins were separated using SDS-PAGE and transferred to a nitrocellulose membrane at 10 V for 40 min. The membranes were blocked in 1% (weight/vol) casein/Tris-buffered saline (TBS) overnight at 4°C in a sealed bag. Following this, the membrane was exposed to the appropriate antibody diluted in 1% casein/TBS at varying concentrations overnight at 4°C, and then washed three times for 10 min each in 1% (weight/vol) casein/TBS. The membrane was then exposed to anti-mouse horse radish peroxidase conjugated antibody, diluted at 1:2,000 in 1% (weight/vol) casein/TBS, for 2 hours at room temperature. The membrane was then washed twice in 1% (weight/vol) casein/TBS for 10 min and, finally, in TBS Tween 20 solution twice for 15 min each. A Roche BM chemiluminescence Blotting substrate POD kit (Roche, Indianapolis, IN, USA) was used, in accordance with the manufacturer's instructions, and placed on membranes for 2 min, after which the membranes were placed inside a LAS-3000 Fuji Film intelligent dark box. The illuminated bands were detected and the image captured using Image reader LAS-3000 software. For each result given, Western blot analysis was performed on three separate occasions with new cell lysates.

To re-probe membranes and analyze protein loading, membranes were stripped with a re-blot solution (Re-blot plus strong; Chemicon International Inc.) at a 1:10 dilution, in accordance with the manufacturer's protocol. The membrane was then blocked with 1% (weight/vol) casein/TBS for 1 hour at room temperature and re-probed with anti-β-actin (1:5,000 in 1% casein/TBS) as a control for protein loading and processed, as described above, as a control for protein loading.

### Densitometry

To determine the density of the bands present in Western blots, a Multi-Gauge V2.3 program (FUJIFILM Medical Systems Inc., Stamford, Connecticut, USA) was used to read band densities. Data were processed, standard deviations calculated, and *t*-tests performed using Microsoft Excel software.

### Indirect immunocytochemistry of cultured cells

Once confluent, cells cultured on glass cover slips were washed three times with PBS, fixed in 4% (weight/vol) paraformaldehyde (PFA/PBS; Sigma-Aldrich) for 10 min at room temperature, and washed again three times in PBS. To permeabilize the cells, 0.1% (vol/vol) Triton X-100/PBS (Amresco Inc., Solon, OH, USA) was applied to cells for 10 min at room temperature, after which the cells were washed three times in PBS and incubated with 1% (weight/vol) bovine serum albumin in PBS (BSA/PBS; Sigma-Aldrich) for 2 hours at room temperature to reduce nonspecific binding of primary antibodies. Following this, 30 μl of the appropriate primary antibody, diluted in 1% (weight/vol) bovine serum albumin/PBS was placed on each cover slip, and the cover slips incubated overnight at 4°C. Cover slips were then washed three times at 15 min each with PBS and were incubated with 30 μl of the secondary antibody (Alexa goat anti-mouse IgG) diluted 1:2000 with 1% (weight/vol) bovine serum albumin/PBS, for 2 hours at room temperature. Cover slips were then washed three times for 5 min each with PBS, and stained with ethidium bromide (diluted 1:10,000 in PBS; Sigma-Aldrich) for 3 to 4 min to visualize nuclei. A final series of three PBS washes of 5 min each was then performed, after which the cover slips were dried and mounted on 1.5 μl of Bio-Rad FluoroGuard™ Anti-fade Reagent (Bio-Rad, Hercules, CA, USA) and cover slip edges sealed with nail polish.

For cells cultured on EHS gel, the filter was inverted and the membrane cut into six to eight segments using a razor. Segments were placed in 35 mm sterile Petri dishes and gently washed three times for 5 min each with PBS. The same procedure was followed for indirect immunocytochemistry, as previously described.

### Microscopy

Cells were viewed using a Leica TCS SP2 AOBS laser scanning confocal microscope (Leica, New South Wales, Australia), using oil immersion and a 40× or 100× objective. Images were captured by the photomultiplier tube using the Leica TCS SP2 laser, and viewed on a workstation using Leica microsystems TCS SP2 software. Images were presented using Microsoft PowerPoint software.

## Results

### CAFs demonstrate greater expression of myofibroblastic markers than NMFs

The phenotypes of human primary NMFs, obtained from reduction mammoplasty tissue, and human primary mammary CAFs, obtained from minced mammary tumour biopsy tissue, were determined by analysis of protein markers associated with the myofibroblast phenotype.

**Figure 1:**
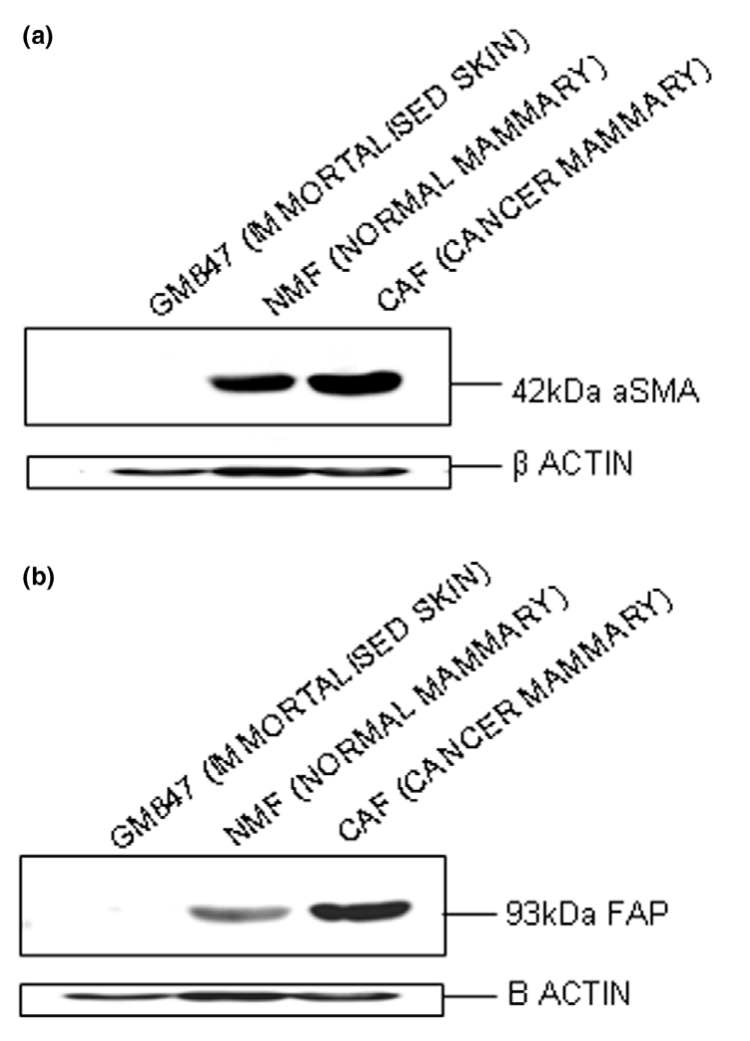
Characterisation of cancer and normal mammary fibroblast lines. **(a) **No α-SMA detected in control GM847 skin fibroblasts (lane 1), but α-SMA was detected in NMFs (lane 2) and in CAFs (lane 3), with an average 2.7-fold increase in band densities for CAFs compared to NMFs. **(b) **FAP was expressed by both NMFs and CAFs (lanes 2 and 3, respectively), but it was not detected in control GM847 skin fibroblasts (lane 1). An average 2-fold increase in band density for CAFs, compared with NMFs, was observed. CAF, cancer-associated fibroblast; FAP, fibroblast activation protein; NMF, normal mammary fibroblast; SMA, smooth muscle actin.

Western blot analysis revealed that α-SMA (Figure 1a) was expressed by both NMFs and CAFs; however, densitometry revealed 2.7-fold higher expression of α-SMA in CAFs as compared with NMFs. A control sample derived from human immortalized skin fibroblasts (GM847) was negative for α-SMA expression (Figure 1a). Western blot analysis indicated that FAP was expressed by both NMFs and CAFs, but not by the GM847 control fibroblasts (Figure 1b). An increase in band density by twofold was seen for CAFs as compared with NMFs (Figure 1b).

### Fibroblasts cause changes in key myoepithelial- and luminal epithelial-specific protein expression by PMC42-LA organoids

PMC42-LA cells cultured on ECM gel coated filters to form organoids with fibroblasts beneath the filter or fibroblast-conditioned medium were analyzed for myoepithelial-specific and luminal epithelial-specific protein markers.

**Figure 2:**
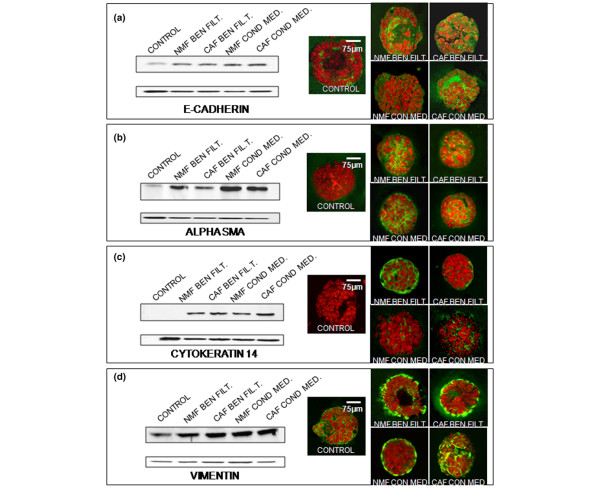
Effect of fibroblasts on major protein expression by PMC42-LA. Western blot analysis for a range of markers on control PMC42-LA organoids (control), and organoids cultured with NMFs beneath the filter (lane 1), with CAFs beneath the filter (lane 2), in medium conditioned by NMFs (lane 3), or in medium conditioned by CAFs (lane 4). Controls had no fibroblasts or fibroblast conditioned-medium. Immunocytochemistry was performed to view organization within organoid structures, and ethidium bromide was used to stain nuclei (red). **(a) **E-cadherin was not significantly upregulated in any of the fibroblast conditions, with no obvious changes in organization. **(b) **α-SMA expression was found to be significantly upregulated by all fibroblast conditions, with no obvious changes in organization. **(c) **Cytokeratin 14 expression was found to be significantly induced by all fibroblast conditions, with no obvious changes in organization. **(d) **Vimentin expression was also found to be significantly upregulated in all fibroblast conditions, with slight changes in organoid organization detected by immunocytochemistry. In control PMC42-LA cells (control lane in panel d), intermediate filament protein vimentin is localized in the cytoplasm of cells throughout organoids. With NMFs beneath the filter (panel d lane 1), vimentin remains localized in the cytoplasm of PMC42-LA cells and vimentin-positive cells are more organized, visible only on outer layer of organoids. With CAFs beneath the filter (panel d lane 2), the same organizational changes are observed. With NMF-conditioned media (panel d lane 3), the same organizational changes are observed. With CAF-conditioned medium (panel d lane 4), no change in organization is observed, with vimentin expression throughout organoids. CAF, cancer-associated fibroblast; NMF, normal mammary fibroblast; SMA, smooth muscle actin.

Compared with control, expression of the luminal epithelial marker E-cadherin, as measured by Western blot analysis, was not significantly upregulated by PMC42-LA organoids cultured with either NMFs (fold increase: 1.8 ± 1.17) or CAFs (fold increase: 1.8 ± 1.31) beneath the filter (Figure 2a, lanes 1 and 2, respectively). Similar values were seen when fibroblast-conditioned medium was used in place of the fibroblasts (fold increase compared with control: 1.5 ± 2.13 with NMF-conditioned medium and 2.4 ± 1.10 with CAF-conditioned medium; Figure 2a, lanes 3 and 4). Immunocytochemistry confirmed that E-cadherin was expressed by most cells in all conditions tested, with no obvious changes in the organization of E-cadherin expressing cells within organoid structures (Figure 2a, lanes 1 to 4).

In the absence of fibroblasts or their conditioned medium, α-SMA was not expressed by PMC42-LA organoids (Figure 2b). When cultured with NMFs beneath the filter, PMC42-LA organoids exhibited a significant 2.5 (± 1.16)-fold increase in α-SMA (*P *< 0.01), similar to that seen in PMC42-LA cultures with CAFs beneath the filter (fold increase: 2.7 ± 0.42, *P *< 0.01; Figure 2b, lanes 1 and 2, respectively). Compared with control, conditioned medium from NMFs caused a significant 6.7 (± 2.76)-fold increase in α-SMA expression (*P *< 0.01) in PMC42-LA cells, which was similar to the significant 7.4 (± 1.20)-fold increase seen in PMC42-LA cells (*P *< 0.001) cultured in CAF-conditioned medium (Figure 2b, lanes 3 and 4, respectively). Immunocytochemistry revealed that α-SMA-expressing cells were organized similarly within organoids in all fibroblast treatment conditions (Figure 2b, lanes 1 to 4).

Cytokeratin 14 was not expressed by PMC42-LA organoids in the absence of fibroblasts/conditioned medium (Figure 2c). However, expression of cytokeratin 14 was induced significantly by the presence of either NMFs (fold increase: 11.5 ± 9.1, *P *< 0.01) or CAFs (fold increase: 12.3 ± 9.3, *P *< 0.01) beneath the filter (Figure 2c, lanes 1 and 2, respectively). Fibroblast-conditioned medium induced expression of cytokeratin 14 similarly, with a significant 10.9 (± 8.8)-fold increase by PMC42-LA in NMF-conditioned medium (*P *< 0.01), and a 14.4 (± 10.2)-fold increase by cells in CAF-conditioned medium (*P *< 0.01; Figure 2c, lanes 3 and 4, respectively). Immunocytochemistry confirmed no obvious changes in the organization of cytokeratin 14-expressing cells within organoids in all fibroblast treatment conditions (Figure 2c, lanes 1 to 4).

### Fibroblasts induce upregulation of vimentin and a change in the orientation of vimentin-positive cells in PMC42-LA organoids

Compared with control, vimentin expression was significantly increased in cells cultured with NMFs beneath the filter (fold increase: 3.9 ± 2.50, *P *< 0.001) and in cells with CAFs beneath the filter (fold increase: 4.4 ± 2.70, *P *< 0.001; Figure 2d, lanes 1 and 2, respectively). In the presence of NMF-conditioned medium, vimentin expression was significantly upregulated by 3.2 (± 1.30)-fold (*P *< 0.001), which was similar to the 3.3 (± 1.80)-fold (*P *< 0.001) increase by cells in CAF-conditioned medium (Figure 2d, lanes 3 and 4, respectively) compared with control. In control PMC42-LA organoids, vimentin-positive cells were distributed throughout the organoids with no apparent pattern in organization (Figure 2d, control). However, when cells were cultured with NMFs beneath the filter, vimentin-positive cells became more organized, and were only visible on the outer layer of organoids (Figure 2d, lane 1). This was also the case when cells were cultured with CAFs beneath the filter (Figure 2d, lane 2) and in NMF-conditioned medium (Figure 2d, lane 3). Surprisingly, PMC42-LA cells cultured in CAF-conditioned medium appeared to form organoids with the same organization as control cells, with vimentin-positive cells being dispersed throughout organoids (Figure 2d, lane 4).

### Fibroblasts induce an intracellular change in localisation of E-cadherin in PMC42-LA cells away from the cell junctions

**Figure 3:**
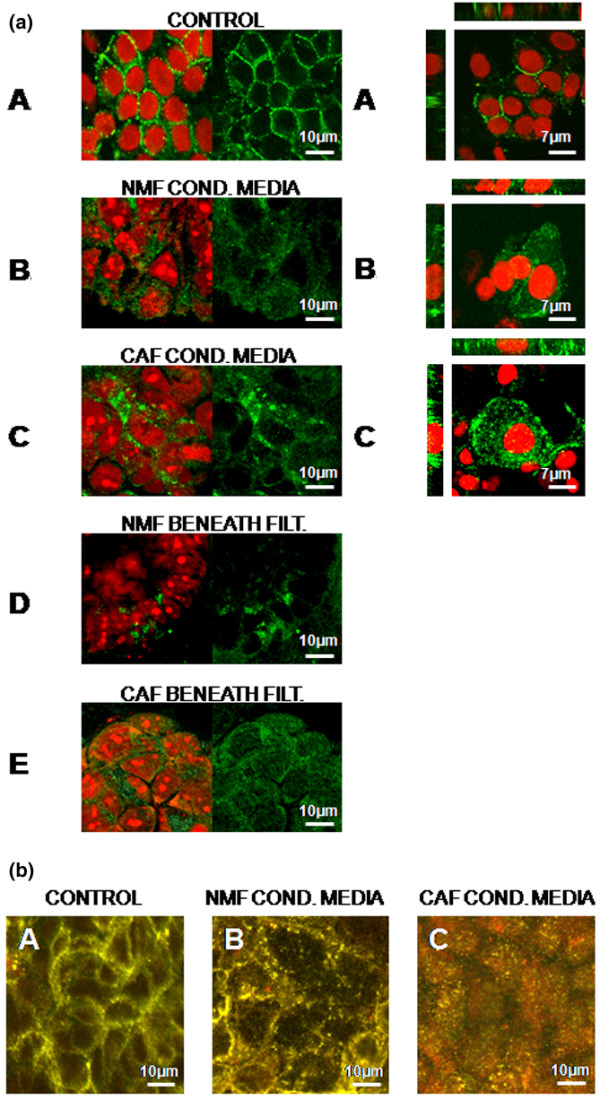
Effect of fibroblasts and conditioned media on E-cadherin and β-catenin (co)localization in PMC42-LA organoids. **(a) **Using immunocytochemistry and confocal microscopy, localization of E-cadherin was analyzed. Nuclei were visualized using ethidium bromide (red). (Part A) In control PMC42-LA organoids (no fibroblasts or conditioned media), E-cadherin staining was observed at cell junctions, which was confirmed by confocal microscopy sectioning of PMC42-LA in 2-dimensional culture (right panel). (Part B) In NMF-conditioned medium E-cadherin label was detected as both junctional and cytoplasmic in PMC42-LA organoids, as confirmed by confocal microscopy sectioning of PMC42-LA in 2-dimensional culture containing NMF-conditioned media (right panel). (Part C) In CAF-conditioned media E-cadherin was also detected as junctional and cytoplasmic in PMC42-LA organoids, with more predominant cytoplasmic localization. This was confirmed in 2-dimensional PMC42-LA culture containing CAF-conditioned media by confocal microscopy sectioning (right panel). With (part D) NMFs beneath filter or (part E) CAFs beneath the filter, E-cadherin was again detected at cell junctions and within cytoplasm. **(b) **E-cadherin and β-catenin are indicated by green and red label, respectively. Areas of colocalization appear yellow. (Part A) In control PMC42-LA cells (no fibroblasts or conditioned media), E-cadherin and β-catenin colocalized at cell junctions with some areas of non-colocalization. (Part B) When in NMF-conditioned medium, colocalization was detected at cell junctions with some independent localization. (Part C) In CAF-conditioned medium, E-cadherin localized to cytoplasm and β-catenin to cytoplasm and nuclei, with some overlap. CAF, cancer-associated fibroblast; NMF, normal mammary fibroblast.

In the absence of fibroblast influences, E-cadherin appeared to have a junctional localization in PMC42-LA cells (Figure 3a part A, left and right panels). The presence of either NMFs or CAFs beneath the filter, or their respective conditioned media, induced a change in E-cadherin localization from cell-cell junctions (Figure 3a part A, left and right panels) to cytoplasm, where it adopted a granular distribution (Figure 3a parts B to E). To better visualize this change in localization within individual cells and obtain sections through cells, PMC42-LA monolayers cultured in fibroblast conditioned medium were also immunostained for E-cadherin; confocal sections through these cells clearly demonstrate some junctional and granular cytoplasmic localization in cells cultured in NMF conditioned medium (Figure 3a part B, right panel). Significantly, the E-cadherin localization appeared more cytoplasmic in PMC42-LA cells in the presence of CAF-conditioned medium (Figure 3a part C, right panel).

### CAF-conditioned medium causes disruption of the cadherin-catenin complex in PMC42-LA cells

In the absence of fibroblast-conditioned media, E-cadherin and β-catenin colocalized to the cell junctions in PMC42-LA monolayer cells (yellow in Figure 3b part A), with sparse areas of independent localization (red/green). When PMC42-LA cells were cultured in NMF-conditioned medium, this colocalization was still apparent (yellow in Figure 3b part B), with some areas of non-colocalization (green/red). However, in the presence of CAF-conditioned medium, β-catenin exhibited a predominantly cytoplasmic label, with some nuclear label (red in Figure 3b part C), with E-cadherin also localised to the cytoplasm (green/yellow in Figure 3b part C). This was performed on PMC42-LA monolayer cultures to allow one to visualize clearly the localization of these two proteins within individual cells.

### CAFs may stimulate more rapid proliferation of PMC42-LA cells than NMFs

Using total cell counts at 24 hours, 3 days and 5 days, some differences in rates of proliferation of PMC42-LA cells in relation to fibroblast-conditioned medium were seen (data not shown). No significant difference was detected at 24 hours, but at 3 days numbers of PMC42-LA cells in CAF-conditioned medium were considerably greater than numbers of PMC42-LA cells cultured in NMF-conditioned medium. At 5 days, this difference was still evident although not significant. This pattern was also seen in three-dimensional culture, with no difference in PMC42-LA proliferation in NMF-conditioned media or CAF-conditioned media at 24 hours; considerably higher cell numbers in CAF-conditioned media at 3 days; and slightly higher, but not significant, proliferation of PMC42-LA in CAF-conditioned medium at 5 days compared with NMF-conditioned medium (data not shown).

### Effects of CAFs and NMFs on cell viability and apoptosis of PMC42-LA cells do not differ

**Figure 4:**
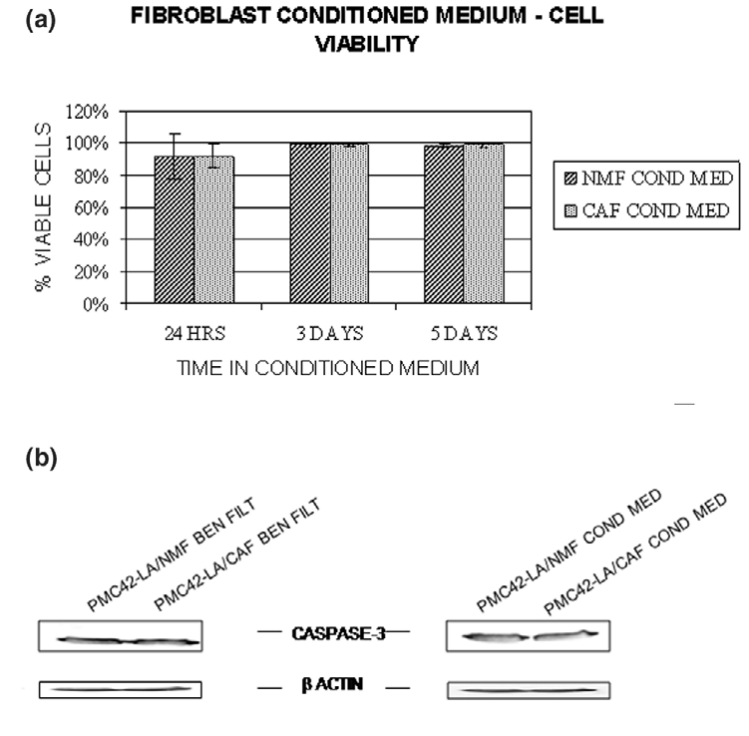
Effect of fibroblast conditioned on apoptosis and cell viability. For PMC42-LA cells cultured in fibroblast-conditioned medium, trypan blue dye exclusion was used to analyze cell viability, and total number of cells were counted and averaged to determine the average percentage of viable cells. **(a) **There was a slight variation in cell viability at 24 hours (75–100% viable cells [average 91.67 ± 14.43% in NMF-conditioned medium] and 85–100% [91.90 ± 7.33%] in CAF-conditioned medium). Similar cell viability was observed at 3 days (97–100% [99.21 ± 1.37%] in NMF-conditioned medium and 98–100% [average 99.40 ± 1.05%] in CAF-conditioned medium). There was no difference at 5 days (97–98% [98.48 ± 0.69%] in NMF-conditioned medium and 97–100% [98.93 ± 1.13%] in CAF-conditioned medium). **(b) **There was no significant difference in active caspase-3 expression between cultures. CAF, cancer-associated fibroblast; NMF, normal mammary fibroblast.

No differences were seen in viability of PMC42-LA cell cultures in the presence of NMFs or CAFs at 24 hours, 3 days and 5 days (Figure 4a), as determined by trypan blue dye exclusion (viable cells calculated as a percentage of total cells counted). Western blot analysis for active caspase-3, a marker of apoptosis, revealed no differences in expression levels for PMC42-LA cells cultured with NMFs beneath the filter as compared with CAFs beneath the filter (densitometry ratio 1:1.2), or in cells cultured in NMF-conditioned medium as compared with those in CAF-conditioned medium (densitometry ratio 1:1.4; Figure 4b).

### CAF-conditioned medium enhances the migratory ability of PMC42-LA cells

**Figure 5:**
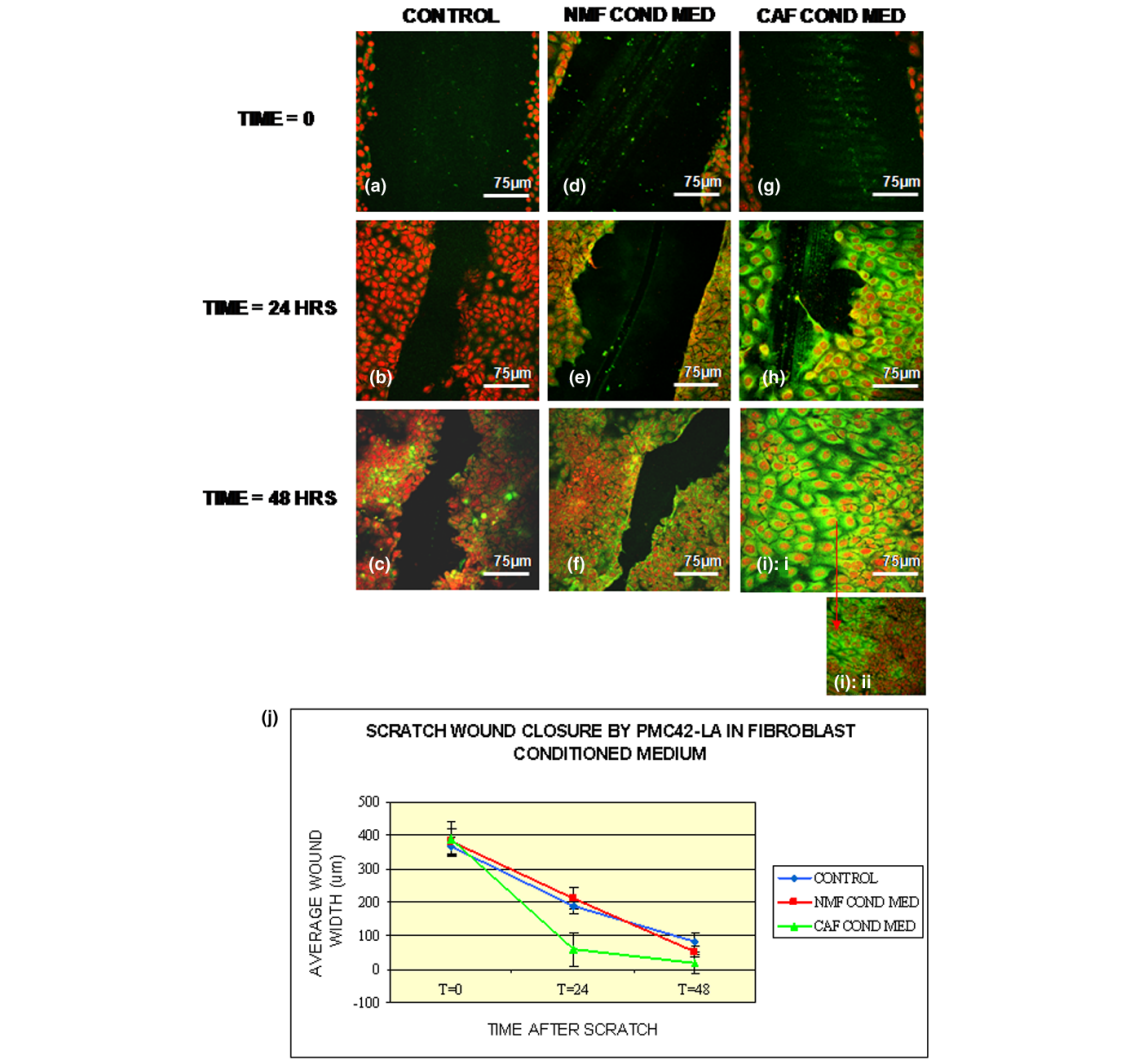
Effect of different fibroblast-conditioned media on cell migration in two-dimensional cultures. PMC42-LA cells were cultured on glass in either normal medium (control), NMF-conditioned medium, or CAF-conditioned medium. Using scratch tests, over a period of 48 hours, PMC42-LA cells were analyzed to determine the rate and extent of wound closure, and vimentin expression. Four scratches averaging between 367 μm (minimum) and 392 μm (maximum) in width were made at time 0. Scratches were measured and averaged, and the extent of wound closure calculated and averaged. **(a-c) **Cells in normal medium (control) exhibited some wound closure at 24 hours (179 ± 21.4 μm), and this had progressed by 48 hours (106 ± 29.0 μm) with small, sparing vimentin expression appearing at 48 hours. **(d-f) **Cells in NMF-conditioned medium exhibited similar rates of wound closure at 24 hours (171 ± 32.9 μm) and 48 hours (160 ± 15.6 μm), with vimentin expression appearing at wound edges at 24 hours. **(g-i) **Cells in CAF-conditioned medium exhibited an accelerated wound closure rate, with the width of scratches reduced by 24 hours (333 ± 52.0 μm) and almost full wound closure by 48 hours (panel i part i: 41.63 ± 28.8 μm). Vimentin expression in these cells was abundant around wound area. (Panel i part ii) Closed wounds were identified at 48 hours by visualizing areas of elongated, vimentin-profuse cells in apparently normal areas of lesser vimentin expression. **(j) **Graphical representation of wound closure by these cultures. CAF, cancer-associated fibroblast; NMF, normal mammary fibroblast.

PMC42-LA cells were cultured on glass to enable the scratch removal of cells and the measurement of wound closure over a period of 48 hours. In the presence of NMF-conditioned medium, PMC42-LA cell wound closure was similar to that in control medium after 48 hours (Figure 5 panels d to f versus panels a to c). Cells cultured in CAF-conditioned medium exhibited rapid wound closure, with most wounds being completely closed by 48 hours (Figure 5 panels g, h and i part ii), visible only by the areas of intense vimentin label among areas of less label (Figure 5i part ii). When the widths of the wounds were measured and averaged, control cells had wounds of approximately 367 ± 29 μm at 0 hours, 188 ± 21 μm at 24 hours and 81 ± 29 μm at 48 hours. Cells in NMF-conditioned medium had wounds of approximately 383 ± 38 μm at 0 hours, 213 ± 33 μm at 24 hours and 52 ± 16 μm at 48 hours, and cells cultured in CAF-conditioned medium had wounds of approximately 392 ± 52 μm at 0 hours, 58 ± 52 μm at 24 hours and 17 ± 29 μm at 48 hours (Figure 5j). Vimentin expression in control cells did not appear changed at 24 hours and was not increased until 48 hours after wound infliction (Figure 5c), whereas cells cultured in the presence of NMF-conditioned medium exhibited a slight increase in vimentin expression by 24 hours (Figure 5e). Cells cultured in CAF-conditioned medium appeared to express more vimentin at 24 and 48 hours (Figure 5h and 5i part i), as compared with control cells and cells cultured in NMF-conditioned medium. In addition to this, some cells in CAF-conditioned medium cultures exhibited an elongated/spindle shape, with some detached cells, neither of which were seen in control or NMF cultures (Figure 5h and 5i part ii).

### Concentration of NMF-conditioned media enhances the migratory ability of PMC42-LA

**Figure 6:**
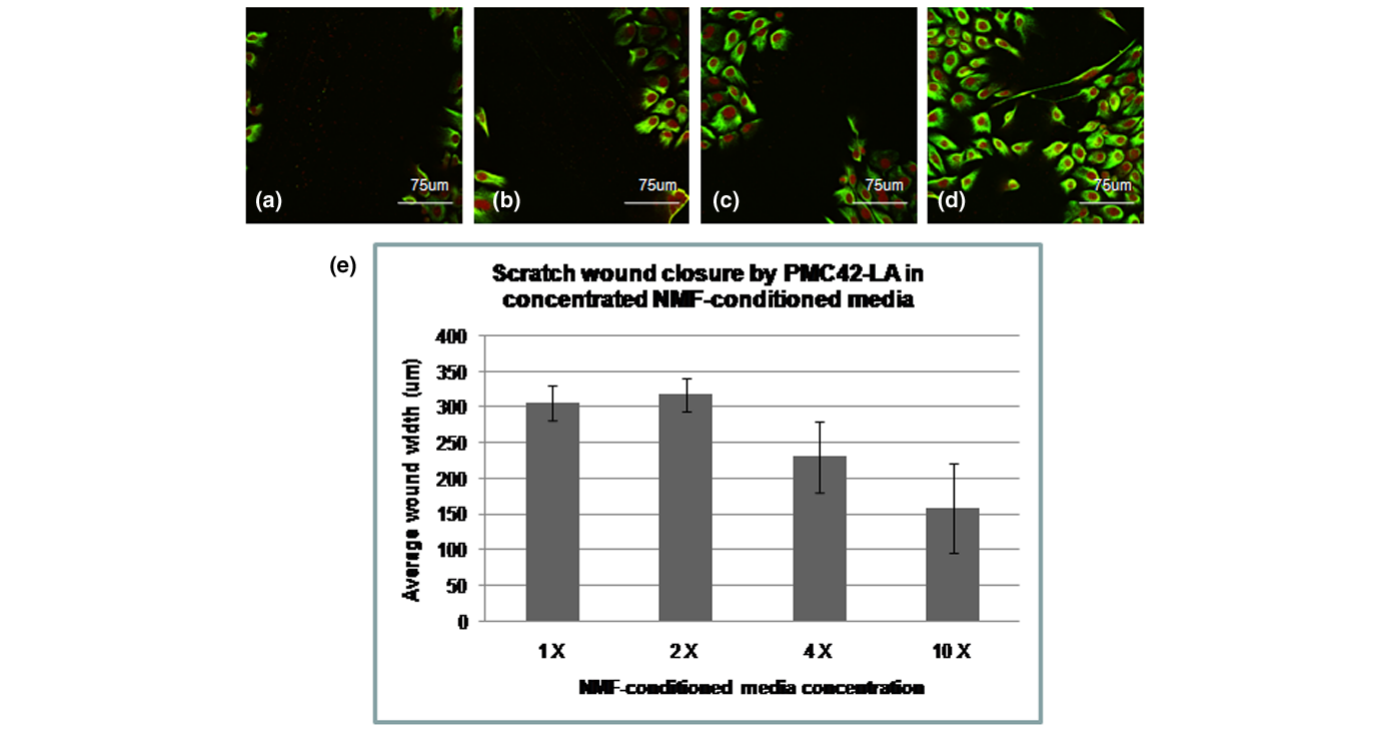
The effect of concentrated NMF-conditioned media on two-dimensional scratch wound closure. PMC42-LA cells were cultured on glass in 1×, 2×, 4×, or 10× NMF-conditioned medium. Using scratch tests, PMC42-LA cells were analyzed for the extent of wound closure 24 hours after scratch. Scratches were measured and averaged, and the extent of wound closure calculated and averaged. **(a) **Cells in 1× NMF-conditioned media (control) had average wound sizes of 307 ± 24.3 μm 24 hours post-scratch. **(b) **Cells in 2× NMF-conditioned medium had average wound sizes of 319 ± 23.2 μm 24 hours post-scratch. **(c) **Cells in 4× NMF-conditioned medium had average wound widths of 232 ± 49.7 μm 24 hours post-scratch, and **(d) **cells in 10× NMF-conditioned medium had average wound widths of 160 ± 62.4 μm 24 hours post-scratch. Vimentin localization appeared unchanged in all cultures; some change in cell morphology was noted in 10× NMF-conditioned media cultures. CAF, cancer-associated fibroblast; NMF, normal mammary fibroblast.

PMC42-LA cells were cultured on glass to enable the scratch removal of cells and the measurement of wound closure 24 hours after scratch. In the presence of NMF-conditioned medium at a 1× concentration/control, PMC42-LA cell wounds at 24 hours averaged 307 ± 24.29 μm (Figure 6a). Cells cultured in a 2× NMF-conditioned medium had an average wound width of 319 ± 23.15 μm (Figure 6b) at 24 hours. Cells cultured in a 4× NMF-conditioned medium had wounds of 232 ± 49.65 μm (Figure 6c) at 24 hours and cells cultured in a 10× NMF-conditioned medium had wounds of 160 ± 62.43 μm (Figure 6d) 24 hours post-scratch. Vimentin localization did not appear changed in any of the concentrated media compared with control, but PMC42-LA cells cultured in 10× NMF-conditioned medium cultures exhibited an elongated/spindle shape, with some detached cells, neither of which were seen in the less concentrated NMF-conditioned media cultures (Figure 6d versus a to c). Average wound widths of these cultures are displayed graphically (Figure 6e).

### CAF-conditioned medium causes budding and detachment of single cells from PMC42-LA organoids in three-dimensional culture

**Figure 7:**
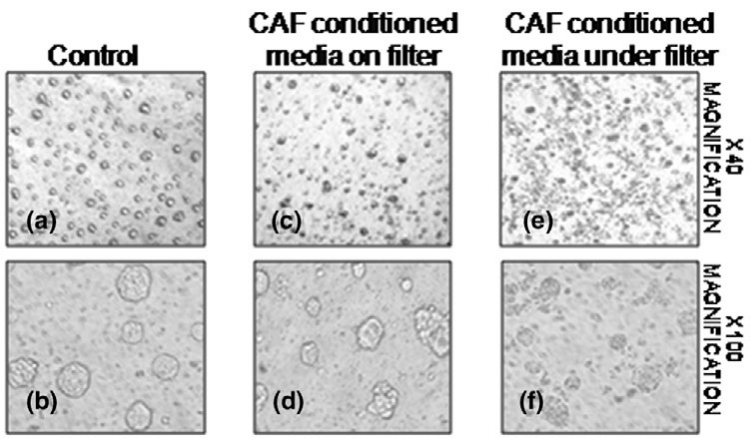
The effect of fibroblast-conditioned media on PMC42-LA organoid morphology in three-dimensional cultures. CAF-conditioned medium was added to or below PMC42-LA filter cultures, and the cultures analysed for changes in organoid morphology possibly representative of increased invasiveness. Controls had no fibroblast conditioned-medium. **(a,b) **In control cultures, organoids appeared spherical with little, if any, single cells present. **(c,d) **With CAF-conditioned media on the filter/culture, organoids remained predominantly spherical, with some budding edges and the presence of single cells and clusters of single cells. **(e,f) **With CAF-conditioned medium below the filter/culture, organoids appeared less spherical, with uneven budding edges and many single cells and clusters of single cells. CAF, cancer-associated fibroblast.

When cultured in control three dimensional-culture, PMC42-LA cells formed round organoids, with few or no single cells present in the culture (Figure 7a,b). When cultured in three-dimensional culture with CAF-conditioned medium, PMC42-LA organoids remained predominantly spherical, although the appearance of some budding edges on organoids and the presence of some single cells in the cultures were noted (Figure 7c,d). Furthermore, when PMC42-LA cells were grown in three dimensional culture with CAF-conditioned medium placed as a chemoattractant beneath the filter insert, organoids once again exhibitied budding edges, in addition to many single cells and clusters of single cells present among the organoids (Figure 7e,f).

## Discussion

In our study we used a human mammary carcinoma cell line with stem cell-like properties [24]* in vitro *to illustrate the differential effects of NMFs and CAFs on EMT. Darcy and coworkers [29] used a similar transwell co-culture system to show that mammary fibroblasts beneath the filter insert were able to stimulate mammary epithelial cell growth and induce alveolar morphogenesis. A similar study conducted by Gache and colleagues [3] also demonstrated that the effect of fibroblasts on co-cultured epithelial cells is via paracrine exchange mechanisms. We extended this approach to compare the effects of fibroblasts derived from malignant mammary tissue (CAFs) with those derived from normal mammary tissue (NMFs). To our knowledge, this is a novel report of selective and direct effects of CAFs on EMT parameters.

NMFs and CAFs can be distinguished on the basis of their differential maker expression. FAP is a 93 kDa cell surface antigen of reactive tumour stromal fibroblasts that is not detected by immunocytochemistry in normal fibroblasts [30-33]. Expression of FAP was seen in both NMFs and CAFs but at a higher level (approximately twofold) in CAFs. To date, there have been no quantitative data published on FAP expression, only studies indicating that resting fibroblasts in normal tissue lack detectable FAP expression by immunocytochemistry [32,33]. This suggests that a low FAP level, undetectable by immunocytochemistry, may be expressed by NMFs and detectable only by Western blot analysis. We detected higher levels of α-SMA in CAFs than in NMFs, confirming the myofibroblastic nature of CAFs. The NMFs used in this study exhibit some α-SMA expression, despite being derived from normal human mammary tissue and, expectedly, the CAFs displayed higher expression (2.7-fold) of this protein. It has been shown that 10% to 80% of cultured human mammary stromal cells synthesize α-SMA after 4 to 11 days in culture, suggesting that elements of smooth muscle differentiation may arise during cell culture of non-smooth-muscle stromal cells that have been taken directly from human breast tissue [34]. Therefore the expression of some α-SMA by NMFs may be an artefact of culture conditions and is not entirely unexpected.

The most significant observation of this study was the differential effects of NMFs and CAFs on the migration of PMC42-LA cells, directly implicating a role for CAFs in inducing cell motility. Following wound infliction in two-dimensional culture, CAF-conditioned medium caused PMC42-LA cells to migrate and upregulate vimentin at the site of the wound at a quicker rate than was seen in control cells or in cells cultured in medium conditioned by NMFs. Wounded control PMC42-LA cells were accompanied by very little vimentin upregulation and incomplete wound closure over 48 hours, similar to PMC42-LA cells cultured in NMF-conditioned medium. The monolayer of control and NMF-conditioned medium cultures remained intact, with no detached cells or changes in cell morphology. In contrast, within 24 hours, PMC42-LA cells cultured in CAF-conditioned medium exhibited a dramatic upregulation of vimentin, with small numbers of detached 'stray' cells. In CAF-conditioned medium, at 24 hours, stray PMC42-LA cells and cells surrounding the wound possessed a spindle-shaped morphology, with some cells exhibiting vimentin-rich protrusions. By 48 hours, almost all PMC42-LA wounds in CAF-conditioned medium were completely closed and could only be visualized by reference to areas of intense vimentin staining and cells with an elongated morphology, stretching in the direction of wound closure. The addition of CAF-conditioned medium to three-dimensional PMC42-LA organoid cultures or below the filters caused 'budding' on some organoids, and the appearance of stray cells and clusters of stray cells surrounding the organoids, which were not seen in control cultures.

These findings, combined with the apparent loss in cell-cell adhesion and upregulation of vimentin, suggest that PMC42-LA are more susceptible to EMT in the presence of CAFs. A slight increase in PMC42-LA migration was noted in NMF-conditioned media cultures compared with control, although to a lesser extent than in CAF-conditioned media, indicating that a factor, or factors, secreted by NMFs may be more highly secreted by CAFs. This was confirmed by experiments evaluating the effect of increasing the concentration of NMF-conditioned medium; specifically, we report a concentration-dependant response by PMC42-LA, with more extensive scratch wound closure in more concentrated NMF-conditioned media.

A key finding in our study was the disruptive effect of CAF-conditioned media on cell-cell junctions. Exposure of PMC42-LA organoids to either NMFs or CAFs caused a change in localization of E-cadherin, a cell-cell adhesion molecule that is normally located at cell junctions. E-cadherin has been shown to be an invasion suppressor in cell culture systems [35-37], and partial or complete loss of E-cadherin expression correlates with poor prognosis in breast cancer patients [38]. E-cadherin was detected mainly in the cytoplasm of PMC42-LA cells after exposure to either type of fibroblast or fibroblast-conditioned medium, and this cytoplasmic localization was more pronounced in PMC42-LA cells cultured in the presence of CAFs than in the presence of NMFs. An abnormal cytoplasmic distribution of E-cadherin has been reported in 17% of invasive lobular breast tumours, with variable expression on cell membranes [39]. The *in vitro *movement of E-cadherin from cell junctions to the cytoplasm has been reported as a consequence of EMT *in vitro *[40]. To our knowledge, these data provide the first evidence that myofibroblasts can induce an EMT in mammary carcinoma cells.

The loss of cellular junctional integrity was confirmed by observations that CAF-conditioned medium induced mis-localization of β-catenin to cytoplasm and nuclear regions in PMC42-LA cells. β-Catenin is an essential component of junctional complexes, linking E-cadherin to the actin filaments [41]. Mouse studies of colorectal cancer have described increased cytoplasmic expression and nuclear localization of β-catenin in chemically induced tumours [42]. E-cadherin has also been shown to recruit β-catenin to the cell membrane and prevent its nuclear localization in SW480 colon carcinoma cells [43]. Plasma membrane-associated staining of E-cadherin and β-catenin has reportedly been absent in invasive lobular carcinomas of the breast [41]. Accumulation of β-catenin in the cytoplasm and nucleus has been hypothesized to promote malignant transformation and progression in breast cancer [44]. Thus the CAF-conditioned medium induced mis-localization of β-catenin seen in our study indicates a substantial role for CAFs in EMT and potentially a role in cancer progression.

Further evidence of an EMT is the upregulation of vimentin by PMC42-LA cells cultured in CAF-conditioned medium. Induction of vimentin is a hallmark of EMT, with almost universal upregulation of this protein [45]. Vimentin is selectively expressed in invasive human breast cancer cell lines [46], reflecting the end-stage of tumour de-differentiation [47,48]. Vimentin is a marker for the mesenchymal phenotype, and upregulation of vimentin may be indicative of EMT [49-51]. Increased expression of vimentin has previously been reported in PMC42-LA cells in response to EGF-induced EMT [26]. Significantly, although the presence of NMFs resulted in the appearance of vimentin-positive cells layered on the outside of organoids, in control organoids and cells cultured in CAF-conditioned medium vimentin-positive cells were localized throughout the organoids.

We postulate that NMFs secrete factors that lead to cellular organization or, conversely, that soluble secreted factors from CAFs disturb cell disorganization. It is possible that that this disorganization was not seen in PMC42-LA cells cultured with CAFs beneath the filter because of insufficient concentrations of these factor(s) being produced by the small number of fibroblasts placed below the filter. A similar loss of polarity and disruption of acinar structures has been reported in three-dimensional HC11 mouse mammary epithelial cell culture, associated with FGF receptor 1 activation. This cellular disorganization was also accompanied by a gain of invasive properties and increased vimentin expression [52]. A hallmark of breast cancer is a loss of polarity and apico-basal organization of epithelial cells, and our results indicate that fibroblasts may have the capacity to alter the organization of epithelial cells within a three-dimensional structure.

Vimentin is not only a marker of EMT but also a marker specific to myoepithelial cells, and therefore upregulation in this protein alone does not suggest EMT. The possibility that CAF-conditioned medium induces a myoepithelial phenotype rather than an EMT can be excluded by the knowledge that both NMFs and CAFs induce these myoepithelial markers, but only CAFs are able to induce migration of PMC42-LA. In addition to an upregulation of vimentin, it is also the separation of cells from the epithelial 'sheets' and a morphologic change to spindle cells that characterises an EMT [53], all of which we report here. *In vitro *studies have shown that soluble factors such as EGF, motility factors and scatter factors promote migratory and locomotive abilities in epithelial cells, accompanied by changes in cell morphology [54-59], thereby promoting an EMT. However, although these factors induce an EMT *in vitro*, no specific *in vivo *counterparts for these growth factors in cancer progression have been found. Our study demonstrates a novel model that mimics the *in vivo *mammary gland, whereby myofibroblasts, through soluble factors, induce breast epithelial cells to undergo an EMT.

In contrast to other studies demonstrating a substantial effect of fibroblasts [60-66] and fibroblast-conditioned medium [67] in stimulating proliferation of cells through paracrine mechanisms [3], in our studies we report only a slight increase in proliferation of PMC42-LA in CAF-conditioned medium compared with NMF-conditioned medium. Over a 5-day period, we report no significant difference in the effect of CAFs compared with NMFs on cell viability or programmed cell death. Other studies have also reported that conditioned medium obtained from fibroblasts derived from both malignant and benign breast tumours had growth stimulatory effects on breast cancer cells, whereas conditioned medium from normal fibroblasts inhibited growth [68]. The extent of proliferation induced may depend upon the source of the fibroblasts, with tumour fibroblasts producing a greater mitogenic response than fibroblasts derived from normal breast tissue [69]. *In vitro *studies using mammary fibroblasts [29] and *in vivo *mouse studies using CAFs and NMFs [70] indicate that the effect of fibroblasts on tumour cells depends upon the type and proportion of inoculated cells. Our study indicates that the key effects of CAFs are on motility, cell organization and EMT marker expression, rather than proliferation.

To ascertain how NMFs and CAFs differentially affect breast epithelial cells, we analyzed PMC42-LA cultured in their conditioned medium for a range of markers. Expression of the myoepithelial marker proteins α-SMA and cytokeratin 14 were significantly upregulated by PMC42-LA organoids upon exposure to both fibroblast types and their respective conditioned medium. Although unrelated to a cancer or EMT phenotype, we previously demonstrated upregulation in myoepithelial-specific proteins by PMC42-LA cells cultured in three dimensions with primary mammary fibroblasts [27]. In the present study, no significant difference in expression levels of myoepithelial proteins was caused by exposure to the different fibroblasts, indicating that both CAFs and NMFs can enrich the myoepithelial population in PMC42-LA culture. A significantly greater increase in α-SMA was seen in cells cultured in either type of fibroblast-conditioned medium, as compared with those with fibroblasts beneath the filter. This may be attributed to the possibility that a greater concentration of fibroblast-secreted soluble factors may be present in fibroblast-conditioned medium, as compared with the less concentrated factors that would be secreted from the small number of fibroblasts beneath the filter.

Arising from the study is the question of the nature of the soluble factors that mediate the differential effects of CAFs and NMFs on mammary epithelial cells. van Roozendaal and coworkers [69] reported that breast fibroblast-conditioned medium contains IGF, which was responsible for MCF-7 proliferation. However, when NMFs and CAFs were compared in terms of IGF-II expression by enzyme-linked immunosorbent assay and real-time polymerase chain reaction, no significant difference was seen [69,71]. Neutralizing antibodies specific to growth factors demonstrated that the growth-stimulatory activity of fibroblasts was not inhibited by anti-IGF-II, anti-EGF, anti-IGF, or anti-TGF-α [72]. Because fibroblasts produce many other growth factors, such as various factors from the hepatocyte growth factor, and basic FGF and IGF families, there are many possibilities and combinations of growth factors that may be responsible for the effect of fibroblasts. Treatments of PMC42-LA cells with IGF-I (10 ng/ml) and IGF-II (10 ng/ml), tumour necrosis factor-α (40 ng/ml), vascular endothelial growth factor (10 ng/ml) and TGF-β (10 ng/ml) failed to replicate the upregulation of vimentin and mis-localization of E-cadherin (data not shown) we found with CAF-conditioned media exposure. Further analysis of the fibroblast-conditioned media is necessary to identify the factors involved; furthermore, the reciprocal effect of cancer cells on the production of growth factors by fibroblasts may need to be taken into account.

## Conclusion

Our study demonstrates a direct role for CAFs in breast cancer progression through the induction of an EMT. Relative to NMFs, CAFs had increased propensity to increase the migratory ability of PMC42-LA cells, commensurate with the characteristics of EMT, including the mis-localization of E-cadherin and translocation of β-catenin, and the upregulation of vimentin. Our study indicates that the factor or factors responsible are secreted by CAFs at a higher level than by NMFs. Elucidation of the mechanism of the cellular interaction between CAFs and mammary epithelial cells will contribute to preventative treatments of breast carcinoma metastasis that act by targeting these factors directly.

## Abbreviations

CAF = cancer-associated fibroblast; DMEM = Dulbecco's modified Eagle medium; ECM = extracellular matrix; EGF = epidermal growth factor; EHS = Engelbreth-Holm-Swarm sarcoma; EMT = epithelial-mesenchymal transition; FAP = fibroblast activation protein; FBS = foetal bovine serum; FGF = fibroblast growth factor; IGF = insulin-like growth factor; NMF = normal mammary fibroblast; PBS = phosphate-buffered saline; SMA = smooth muscle actin; TBS = Tris-buffered saline; TGF = transforming growth factor.

## Competing interests

The authors declare that they have no competing interests.

## Authors' contributions

SCL carried out all experimental studies; MLA and SCL conceived the study. DFN and EWT participated equally in the design of the study and interpretation of the results, and MLA analyzed results extensively and drafted the manuscript. All authors read and approved the final manuscript.
